# Detection and Localization of Rotor Winding Inter-Turn Short Circuit Fault in DFIG Using Zero-Sequence Current Component Under Variable Operating Conditions

**DOI:** 10.3390/s25092815

**Published:** 2025-04-29

**Authors:** Muhammad Shahzad Aziz, Jianzhong Zhang, Sarvarbek Ruzimov, Xu Huang

**Affiliations:** School of Electrical Engineering, Southeast University, Nanjing 210096, China; shahzadaziz@seu.edu.cn (M.S.A.);

**Keywords:** DFIG, inter-turn short circuit fault, zero-sequence current, fault detection

## Abstract

DFIG rotor windings face high stress and transients from back-to-back converters, causing inter-turn short circuit (ITSC) faults. Rapid rotor-side dynamics, combined with the unique capability of DFIG to operate in multiple modes, make the fault detection in rotor windings more challenging. This paper presents a comprehensive methodology for online ITSC fault diagnosis in DFIG rotor windings based on zero-sequence current (ZSC) component analysis under variable operating conditions. Fault features are identified and defined through the analytical evaluation of the DFIG mathematical model. Further, a simple yet effective algorithm is presented for online implementation of the proposed methodology. Finally, the simulation of the DFIG model is carried out in MATLAB/Simulink under both sub-synchronous and super-synchronous modes, covering a range of variable loads and low-frequency conditions, along with different fault severity levels of ITSC in rotor windings. Simulation results confirm the effectiveness of the proposed methodology for online ITSC fault detection at a low-severity stage and precise location identification of the faulty phase within the DFIG rotor windings under both sub-synchronous and super-synchronous modes.

## 1. Introduction

Doubly fed induction generator (DFIG) is a key technology in modern wind power generation and is increasingly considered an optimal choice for pumped-hydro power generation systems. Its advantages include variable speed operation, autonomous control of active and reactive power, and a smaller converter size. However, under harsh environments and complex operating conditions, unanticipated faults in different DFIG components may occur, negatively impacting its performance and reliability [1,2,3,4,5].

DFIG has windings on both the stator and rotor, where inter-turn short circuit (ITSC) faults are among the most common and can occur in either winding. Statistical data indicate that rotor and stator ITSC faults constitute approximately 10% and 40% of all induction machine faults, respectively [6]. DFIG rotor windings face high stress and transients from the rotor-side back-to-back PWM converter, leading to high possibility of ITSC fault occurrence. ITSC faults typically begin with insulation degradation, causing some windings in the short-circuited section to carry extremely high currents. During this stage, the stator and rotor circuits remain undisturbed, allowing the machine to continue operating despite this undetected internal fault. Early detection is therefore crucial to prevent its progression into a full short circuit, which could cause severe system damage [7,8,9,10].

Rotor winding ITSC faults in DFIG have gained research attention due to their potential to cause imbalanced magnetic fields, enhanced vibrations, and excessive heating, leading to severe machine damage if not identified early at a low-severity stage. Compared to stator winding ITSC faults, relatively limited research has focused on detecting rotor winding ITSC faults, primarily due to the rotor-side complex construction and dynamic low frequency. The rotor-side frequency *f_r_* varies with DFIG operating conditions, since it depends on the stator-side frequency *f_s_* and the slip *s* (i.e., *f_r_* = *s* × *f_s_*). Because DFIG typically operates at low slip values, the rotor-side frequency remains low, making fault detection in the rotor winding more challenging under variable speed and load conditions [11].

Many investigations on rotor and stator ITSC faults in DFIG and other electrical machines have been conducted, and various online diagnostic techniques have been proposed [10,12,13]. These techniques analyze a range of electrical and mechanical signals, including current [12,13,14,15], vibration [16], transient leakage flux [17], magnetic airgap flux [18], power [19,20], torque [21], speed [22,23,24], and temperature [25,26,27]. Among these, machine current signature analysis (MCSA) is the most popular method and primarily involves the analysis of current signals obtained directly from the machine terminals. These signals are processed to reveal distinctive anomalies within the frequency spectrum as fault indicators by utilizing the appropriate signal processing techniques, such as Fast Fourier transform (FFT) [28], Discrete Wavelet Transform (DWT) [29], Empirical Mode Decomposition (EMD) [21], and Artificial Neural Networks (ANNs) [30]. The anomalies are compared against typical operational baselines to confirm ITSC faults, enabling targeted maintenance actions to prevent further damage and enhance machine reliability.

Although these techniques have several advantages, including their non-invasive nature and cost-effectiveness, they also have certain limitations. For instance, the harmonic components of the signature signals under an inter-turn fault may be mixed with those present in a healthy winding condition or suppressed by the control system, which reduces the effectiveness of the MCSA method in practical applications [14,31,32]. Additionally, most of these techniques are primarily suited for analyzing steady-state conditions and are highly sensitive to operational dynamics, such as sudden changes in load or speed [13,33]. Consequently, these techniques are not suitable for identifying faults in machines involving variable load and speed operation, such as the DFIG for wind power generation. Furthermore, the DFIG’s large size, complex structure, capability to operate under various slip modes, and necessity for additional measuring equipment while using these techniques pose further challenges and undermine the accuracy of the fault diagnosis process in DFIG.

Embedded controller signals in combination with the machine signature signals have been utilized to overcome the limitations of MCSA-based techniques that primarily analyze signature signals from machine terminals, which can sometimes miss subtleties of certain faults due to signal suppression by the control system or external noise [32]. Embedded control signals, which include data from controller operation like rotor position or error signals between actual and target performance parameters, provide direct insight into the system dynamics and its interaction with the control system. By using these internal signals, diagnostics can achieve higher sensitivity and specificity in detecting and isolating faults, particularly those that do not manifest clearly in external electrical signatures, thus enhancing fault detection under variable operating conditions and reducing dependency on the quality of external signals [34,35]. While embedded control signals are effective for enhancing fault detection in DFIG systems, they typically lack the ability to identify the faulty phase location directly [36,37]. This limitation arises from the fact that these signals, instead of providing phase-specific details, frequently offer a more generalized presentation of the performance and control dynamics of the system. While they excel in diagnosing the type of fault or overall system health, additional analysis or supplementary techniques might be necessary to pinpoint which specific phase is affected by a fault. This phase identification is crucial for targeted maintenance and repair.

The sequence component-based methods are proposed to address the deficiencies in other ITSC fault detection techniques, including embedded signals and MCSA methods. These methods analyze the negative sequence [11] and zero-sequence [38] voltages and currents and have successfully been applied to detect ITSC faults in electric machines, including DFIG. The sequence components are sensitive to machine faults, and their analysis enhances the reliability of the fault detection process [13,39,40,41]. However, these particular methods have notable limitations. Firstly, the amplitude of the sequence components used as fault indicator is highly influenced by variations in the machine rotor speed and reduces the accuracy of fault diagnosis process. Secondly, the topologies used for extracting the fault indicators lack in analytical evaluation and might not be suitable for online use, and finally, the location of the faulty phase is not identified in most of the cases [31,38,42]. The fundamental component (FC) in zero-sequence voltage and current is particularly sensitive to ITSC faults, making it a reliable indicator for fault detection and faulty phase identification, with fault features extracted through the analytical evaluation. However, most of the studies in this area primarily examine cases where machines function under sub-synchronous or synchronous modes with a constant supply frequency [22,42]. Since the DFIGs can operate in super-synchronous mode and exhibit dynamic low-frequency on the rotor-side as the speed varies, consequently, these studies do not fully consider the DFIG dynamics and are not suitable for identifying faults in the DFIG rotor windings.

This paper proposes a comprehensive method based on zero-sequence current (ZSC) component for the ITSC fault diagnosis in DFIG rotor windings. The proposed method can effectively detect the fault and its severity as well as the position of the faulty phase under both sub-synchronous and super-synchronous modes of DFIG operation, while remaining robust under low dynamic rotor-side frequency, variable speed, and load conditions. It can detect the fault at an early stage with a low-severity level. In addition, through the analytical evaluation of the DFIG model with rotor ITSC fault, an algorithm has been proposed that allows for the easy implementation of the online diagnosis method. The validation of the proposed method has been realized through the simulations in MATLAB/Simulink.

The following points highlight the main contributions of the paper:

- This paper analytically validates the rotor winding ITSC fault detection in DFIG under variable operating conditions by using a detailed mathematical model that offers a strong theoretical foundation for practical applications.
- This paper introduces a fault detection algorithm that validates fault detection under both sub-synchronous and super-synchronous modes of DFIG operation with rotor-side dynamic low-frequency conditions, whereas previous research has largely overlooked the super-synchronous mode and dynamic low-frequency conditions.
- This paper uses the FC in rotor currents and ZSC under rotor dynamic frequency for detecting the ITSC fault and identifying the fault location which simplifies the implementation and enhances the sensitivity and accuracy of the rotor winding ITSC fault detection in DFIG.

Table 1 presents a summary of the existing methods in the literature, highlighting the limitations of these existing methods in diagnosing the ITSC faults and the contributions of the proposed method.

Subsequent sections of this paper are structured as follows. In Section 2, a model of DFIG with an inter-turn short circuit in rotor winding is developed. In Section 3, analytical evaluation is conducted, fault indicators are defined, and the proposed fault detection methodology is outlined which relies on utilizing the ZSC component. In Section 4, simulations are carried out and results are presented. In Section 5, discussion is presented. In Section 6, the conclusions are finally drawn.

## 2. Modeling

**Figure 1:**
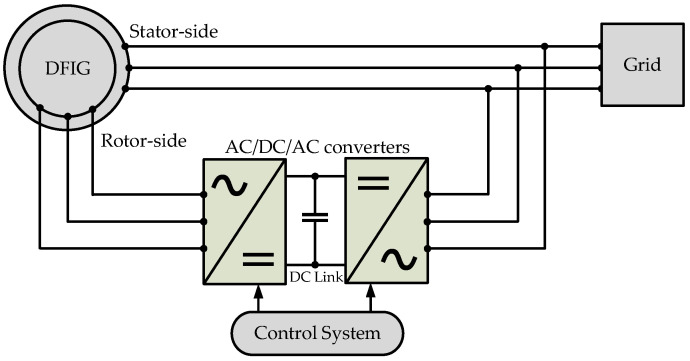
Schematic diagram of the DFIG system.

The variable-speed DFIG employed in wind turbines includes a stator that connects directly to the grid and a rotor equipped with its own three-phase windings. These rotor windings are connected to external circuits via slip rings and brushes, facilitating the independent control of rotor currents. The system incorporates AC/DC/AC converters between the rotor and the grid to adjust the frequency and magnitude of the supply fed to or from the rotor, optimizing the DFIG interaction with the grid. A sophisticated control system manages the converters, ensuring efficient operation across varying speeds and grid demands, making the DFIG highly adaptable and efficient for renewable energy applications. The schematic diagram of the DFIG system is shown in Figure 1.

### 2.1. Inter-Turn Short Circuit Fault

**Figure 2:**
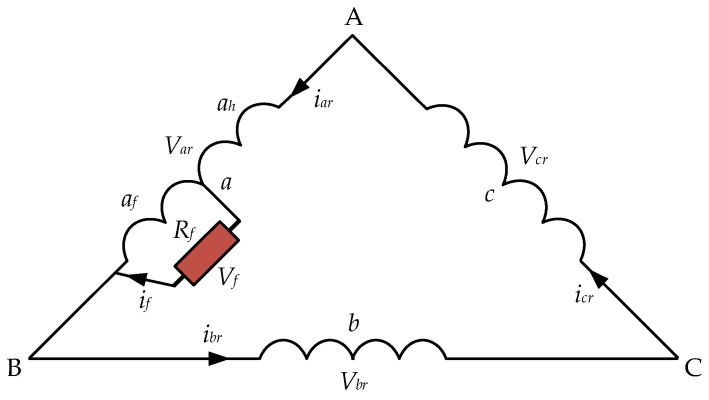
Illustration of ITSC fault in phase ‘*a*’ of rotor windings.

The ITSC fault occurs due to insulation failure in a specific phase of a winding. Figure 2 shows an ITSC fault in phase ‘*a*’ of the delta-connected rotor windings of the DFIG, where *R_f_* represents the fault resistance, *i_f_* represents the fault current caused by ITSC, and *V_f_* denotes the voltage drop due to the fault.

The inclusion of an ITSC fault in phase ‘a’ causes the rotor windings to consist of four phases, as the faulty phase divides into a healthy (‘*a_h_*’) and a faulty (‘*a_f_*’) part. More dynamics are introduced in the DFIG system when more faulty turns are added to the faulty part (‘*a_f_*’) of the rotor winding. *R_f_* decreases, which effectively reduces the overall resistance of the phase ‘a’. Analysis of these dynamics can be a useful tool for fault diagnosis in rotor windings of the DFIG [42,43].

### 2.2. Mathematical Model of DFIG with Rotor ITSC

An abc-reference frame model of DFIG is derived to introduce the ITSC fault in any single phase of the rotor windings. The parameter *µ* represents the fault level and is defined as the ratio between the short-circuited turns *n* and the total turns *N* in the concerned phase, i.e., *n*/*N*. Therefore, the voltage equations suitable for a three-phase DFIG having an ITSC fault in phase ‘*a*’ of the rotor windings can be presented as follows [22,44,45]:

(1) $$\left[\begin{array}{c} V_{abcs}\\ \begin{array}{l} V_{abcr}\\ V_{f} \end{array} \end{array}\right]=\left[\begin{array}{ccc} Rs & 0_{3X3} & 0_{3X1}\\ 0_{3X3} & R_{rf} & 0_{3X1}\\ 0_{1X3} & 0_{1X3} & \mu r_{r} \end{array}\right]\left[\begin{array}{c} i_{abcs}\\ i_{abcr}\\ i_{f} \end{array}\right]+\frac{d}{dt}\left[\begin{array}{c} \phi_{abcs}\\ \phi_{abcr}\\ \phi_{f} \end{array}\right]$$

(2) $$\left[\begin{array}{c} \phi_{abcs}\\ \phi_{abcr}\\ \phi_{f} \end{array}\right]=\left[\begin{array}{ccc} L_{ss} & L_{sr} & \mu L_{1}\\ L_{rs} & L_{rr} & \mu L_{2}\\ \mu L_{1}^{T} & \mu L_{2}^{T} & \mu^{2}L_{r} \end{array}\right]\left[\begin{array}{c} i_{abcs}\\ i_{abcr}\\ i_{f} \end{array}\right]$$

where *V_abcs_* and *V_abcr_* are three-phase voltages in stator and rotor, respectively; *i_abcs_* and *i_abcr_* are three-phase currents in stator and rotor, respectively; *ϕ_abcs_* and *ϕ_abcr_* are magnetic fluxes in stator and rotor, respectively; *R_s_* and *R_rf_* are stator three-phase resistance and rotor three-phase resistance under fault, respectively; and *V_f_*, *i_f_*, and *ϕ_f_* are fault voltage, fault current, and fault-induced magnetic flux, respectively. These quantities are further given as below:

$$\begin{array}{l} \left[V_{abcs}\right]=\left[\begin{array}{ccc} V_{as} & V_{bs} & V_{cs} \end{array}\right];\left[V_{abcr}\right]=\left[\begin{array}{ccc} V_{ar} & V_{br} & V_{cr} \end{array}\right];\left[i_{abcs}\right]=\left[\begin{array}{ccc} i_{as} & i_{bs} & i_{cs} \end{array}\right];\\ \left[i_{abcr}\right]=\left[\begin{array}{ccc} i_{ar} & i_{br} & i_{cr} \end{array}\right];\left[\phi_{abcs}\right]=\left[\begin{array}{ccc} \phi_{as} & \phi_{bs} & \phi_{cs} \end{array}\right];\left[\phi_{abcr}\right]=\left[\begin{array}{ccc} \phi_{ar} & \phi_{br} & \phi_{cr} \end{array}\right]; \end{array}$$

$$R_{s}=\left[\begin{array}{ccc} r_{s} & 0 & 0\\ 0 & r_{s} & 0\\ 0 & 0 & r_{s} \end{array}\right];L_{ss}=\left[\begin{array}{ccc} L_{s} & -\frac{1}{2}M_{s} & -\frac{1}{2}M_{s}\\ -\frac{1}{2}M_{s} & L_{s} & -\frac{1}{2}M_{s}\\ -\frac{1}{2}M_{s} & -\frac{1}{2}M_{s} & L_{s} \end{array}\right];$$

$$R_{rf}=\left[\begin{array}{ccc} (1-\mu )r_{r} & 0 & 0\\ 0 & r_{r} & 0\\ 0 & 0 & r_{r} \end{array}\right];L_{rr}=\left[\begin{array}{ccc} {\left(1-\mu \right)}^{2}L_{r} & -\frac{1}{2}(1-\mu )M_{r} & -\frac{1}{2}(1-\mu )M_{r}\\ -\frac{1}{2}(1-\mu )M_{r} & L_{r} & -\frac{1}{2}M_{r}\\ -\frac{1}{2}(1-\mu )M_{r} & -\frac{1}{2}M_{r} & L_{r} \end{array}\right];$$

$$L_{sr}=L_{rs}^{T}=M_{r}\left[\begin{array}{ccc} (1-\mu )\cos\theta_{r} & \cos(\theta_{r}-\frac{2\pi }{3}) & \cos(\theta_{r}+\frac{2\pi }{3})\\ \left(1-\mu )\cos(\theta_{r}+\frac{2\pi }{3}\right) & \cos\theta_{r} & \cos(\theta_{r}-\frac{2\pi }{3})\\ \left(1-\mu )\cos(\theta_{r}-\frac{2\pi }{3}\right) & \cos(\theta_{r}+\frac{2\pi }{3}) & \cos\theta_{r} \end{array}\right]$$

$$L_{1}={\left[\begin{array}{ccc} (1-\mu )L_{r} & -\frac{1}{2}M_{r} & -\frac{1}{2}M_{r} \end{array}\right]}^{T};\;L_{2}={\left[\begin{array}{ccc} \cos\theta_{r} & \cos(\theta_{r}-\frac{2\pi }{3}) & \cos(\theta_{r}+\frac{2\pi }{3}) \end{array}\right]}^{T}$$

Here, *M_s_* and *M_r_* are stator and rotor mutual inductances, respectively; *L_s_* and *L_r_* are the total inductances of stator and rotor, respectively; and *r_s_* and *r_r_* are the stator and rotor per phase resistances, respectively. Then, the electromagnetic torque equation takes the following form under the ITSC fault condition:

(3) $$T_{e}=P\left(i_{s}^{T}\frac{\partial L_{sr}}{\partial \theta_{r}}i_{r}+\mu f_{x}^{T}i_{f}\frac{\partial L_{sr}}{\partial \theta_{r}}i_{r}\right)$$

The motion equation is given as the following:

(4) $$T_{m}=T_{e}-\left(\frac{J}{n_{p}}\frac{d\omega_{r}}{dt}+\frac{D\omega_{r}}{n_{p}}\right)$$

where *T_m_* and *T_e_*, *D* and *J*, and *ω_r_*_,_ and *n_p_* are electromagnetic and mechanical torques, inertia and damping coefficient of the machine, and angular velocity and number of poles of the machine, respectively.

## 3. Methodology

The proposed methodology outlines the detection technique for rotor winding ITSC fault in DFIG where the stator three-phase winding is connected in star configuration and rotor three-phase winding is connected in delta configuration. The analytical evaluation of the rotor ITSC fault diagnosis using the zero-sequence current (ZSC) is presented.

### 3.1. Expression for ZSC Signal

The delta-connected rotor winding is supplied through a back-to-back power converter. Under normal DFIG operation with a healthy rotor winding, three rotor phases carry equal currents, summing to zero. Consequently, zero-sequence current (ZSC) is ideally absent from the winding.

(5) $$i_{ar}+i_{br}+i_{cr}=0\;\mathrm{and}\;i_{0}=0$$

where *i*_0_ is the ZSC component. Under the abnormal DFIG operation conditions, the currents become unbalanced in the three phases, and their sum is no longer zero which leads to the flow of ZSC in the arms of the delta-connected rotor winding. When ITSC fault occurs in one of rotor windings, the sum of three phase rotor currents is given as follows:

(6) $$i_{ar}+i_{br}+i_{cr}=i_{0}\;\mathrm{and}\;i_{0}>0$$

**Figure 3:**
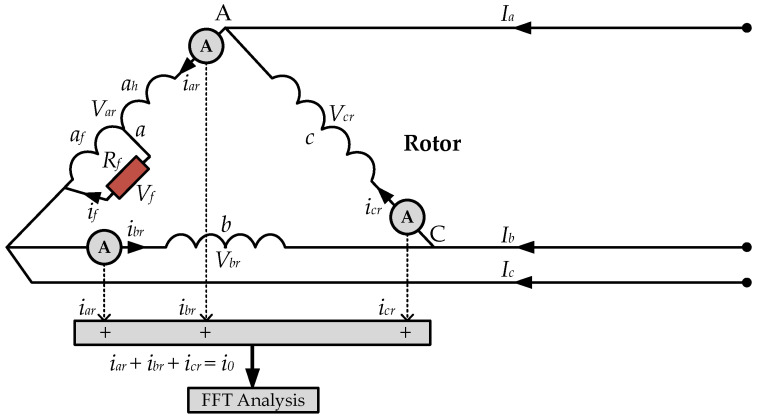
ZSC measurement for delta-connected rotor windings of DFIG.

This ZSC in (6) can serve as a useful signal for providing information about the ITSC fault incident in the rotor windings of the DFIG. Because there is no ZSC in the lines, but it only follows in the phase arms of the delta winding, the ZSC is measured by summing the three phase currents using three current sensors installed one in each phase winding, as shown in Figure 3.

This method provides a clear signal for fault detection purposes by directly isolating the ZSC component from the rotor circuit. As it monitors each phase current separately, it enhances fault detection accuracy by isolating the faults to specific rotor phases. It is simple to implement with measurement instruments and can be adapted for real-time condition monitoring without requiring significant modifications to the existing rotor windings.

By applying Kirchhoff’s Voltage Law (KVL) to the delta-connected rotor windings, the three-phase rotor voltages sum to zero. Hence, referring to Figure 3, it yields the following:

(7) $$V_{ar}+V_{br}+V_{cr}=0$$

By substituting *V_ar_*, *V_br_*, and *V_cr_* from rows 4, 5, and 6 of (1) into (7), it gives the following:

(8) $$\begin{array}{l} 0=(i_{ar}+i_{br}+i_{cr})R_{r}-\mu i_{ar}R_{r}+[\mu (\mu -2)L_{r}+\frac{1}{2}\mu M_{r}]\frac{di_{ar}}{dt}+\\ (L_{r}-M_{r}+\frac{1}{2}\mu M_{r})(\frac{di_{ar}}{dt}+\frac{di_{br}}{dt}+\frac{di_{cr}}{dt})+[\mu (1-\mu )L_{r}-\mu M_{r}]\frac{di_{f}}{dt} \end{array}$$

Under the ITSC fault conditions, the fault current *i_f_* is dominant in the faulty phase which causes the ZSC to flow within the winding. Also, the rate of change of fault current is dominant, while the phase currents exhibit a small and equal rate of change. Therefore, (8) can be simplified as follows:

(9) $$0=(i_{ar}+i_{br}+i_{cr})R_{r}-\mu i_{f}R_{r}+[-\mu L_{r}-\frac{1}{2}\mu M_{r}]\frac{di_{f}}{dt}$$

Substituting (6) into (9), it simplifies to the following:

(10) $$i_{0}=\frac{1}{R_{r}}[\mu i_{f}R_{r}+\mu (L_{r}+\frac{1}{2}M_{r})\frac{di_{f}}{dt}]$$

The expression in (10) gives the ZSC under ITSC fault conditions. It is evident that the ZSC is absent under healthy conditions because *i_f_* is zero. However, *i_f_* is very large and difficult to measure under fault conditions; therefore, it needs to be eliminated from (10). This can be achieved by subtracting row seven of (1) from row four.

(11) $$V_{ar}-V_{f}=(1-\mu )i_{f}R_{r}+(1-2\mu )[(1-\mu )L_{r}\frac{di_{ar}}{dt}+\mu L_{r}]\frac{di_{f}}{dt}$$

Solving (11) for *i_f_*, it gives the following:

(12) $$i_{f}=\frac{\mu i_{ar}R_{r}}{R_{f}+(1-\mu )R_{r}}$$

It is evident from (12) that the fault current *i_f_* carries the same initial phase angle (IPA) as the faulty phase ‘*a*’ current (*i_ar_*) under the ITSC fault condition. By substituting (12) into (10), it yields the following expression:

(13) $$i_{0}=\frac{\mu^{2}}{R_{r}(R_{f}+(1-\mu )R_{r})}[R_{r}^{2}i_{ar}+R(L_{r}+\frac{1}{2}M_{r})\frac{di_{ar}}{dt}]$$

Considering that the ZSC in (13) may contain different harmonics induced from the stator and converter, only the FC in ZSC is adopted for efficient fault diagnosis, which is large and sensitive to ITSC faults. Hence, if only the FC is taken into consideration, *i_ar_* and *i_f_*, as they carry the same IPA, can be expressed as follows:

(14) $$i_{ar}=I_{ar}\sin(\theta +\theta_{iar})$$

(15) $$i_{f}=I_{f}\sin(\theta +\theta_{iar})$$

where *I_ar_* and *θ_iar_* are the FC magnitude and IPA of the current *i_ar_*, respectively. *I_f_* is the magnitude of fault current *i_f_*. Substituting (14) into (13), it yields the following:

(16) $$i_{0}=\frac{\mu^{2}}{R_{r}(R_{f}+(1-\mu )R_{r})}[R_{r}^{2}I_{ar}\sin(\theta +\theta_{iar})+R(L_{r}+\frac{1}{2}M_{r})\frac{d}{dt}I_{ar}\sin(\theta +\theta_{iar})]$$

As IPAs of *i_f_* and *i_ar_* are the same and rest of the factors are constant in (12), only the magnitudes *I_ar_* and *I_f_* of these currents can be considered. Then, combining (12) and (16), the expression simplifies to the following:

(17) $$i_{0}=\frac{\mu I_{f}}{R_{r}}[\sqrt{R_{r}^{2}+\omega_{r}^{2}{\left(L_{r}+\frac{1}{2}M_{r}\right)}^{2}}\sin(\theta +\theta_{iar}+\delta )]$$

It can be observed that (17) comprises the magnitude and the phase angle. Hence, it can be expressed as follows:

(18) $$i_{0}=I_{0M}\sin(\theta +\theta_{i0})$$

where *I*_0*M*_ and *θ_i_*_0_ are the magnitude and IPA of the FC in ZSC, respectively. From (17), they can be expressed as follows:

(19) $$\left\{\begin{array}{l} I_{0M}=\frac{\mu I_{f}}{R_{r}}[\sqrt{R_{r}^{2}+\omega_{r}^{2}{\left(L_{r}+\frac{1}{2}M_{r}\right)}^{2}}]\\ \theta_{i0}=\theta_{iar}+\delta \\ \delta =\tan^{-1}(\omega_{r}(L_{r}+\frac{1}{2}M_{r})/R_{r}) \end{array}\right.$$

where *δ* is the shift angle due to the fault and denotes the difference between the IPAs of the FC in ZSC and the faulty phase current, *i_ar_*, in this case.

### 3.2. Fault Indicator

It can be seen from (19) that the magnitude of the FC in ZSC depends on the ITSC fault severity level *µ* and is, therefore, identified as the indicator for the occurrence of ITSC fault in DFIG rotor windings. The greater the fault severity level, the greater the FC in ZSC, and vice versa. However, it is always necessary to set a threshold value for the ZSC because some level of ZSC always exists in the system due to the measurement noise effects and minor dynamics in the system. This threshold value can be obtained and pre-set by measuring the ZSC under healthy operating conditions of the machine when no ITSC fault is present in the rotor windings. This pre-set threshold will help to avoid false indications for fault detection and only offer the correct signal for fault, i.e., only when the ZSC magnitude exceeds its pre-set threshold value. Considering *I_TH_* as the pre-set threshold value of FC magnitude in ZSC, the condition for an ITSC fault occurrence is given by the following:

(20) $$I_{0M}>I_{TH}$$

To ensure the accuracy of *I*_0*M*_ when determining an ITSC fault under the condition in (20), only the FC component of *I*_0*M*_ is considered, as it is both large and highly sensitive to ITSC faults. The FC is extracted using FFT which effectively filters out higher-order harmonics and noise, improving the reliability of the fault detection. Additionally, the threshold *I_TH_* is established through the multiple measurements under the healthy conditions to account for system noise and minor dynamic variations.

#### Fault Indicator Under Dynamic Conditions

However, since the DFIG can operate in sub-synchronous, synchronous, and super-synchronous modes under variable wind and load conditions, the rotor-side fundamental frequency changes with the rotor dynamic speed. Also, the rotor-side frequency depends on the slip and the stator-side frequency and can be defined as follows:

(21) $$f_{r}=s\times f_{s}$$

where *s* is the slip of DFIG which is primarily determined by the rotor speed and the load. Hence, from (21), the FC on the rotor side can be extracted and analyzed under the DFIG variable load and speed conditions, ensuring that the fault indication remains independent of the machine’s operating conditions.

### 3.3. Fault Position Indicator

It is evident from (19) that the IPA of the FC in ZSC (*θ_i_*_0_) depends on the IPA of the FC in faulty phase ‘*a*’ current (*θ_iar_*) and the shift angle due to fault (*δ*). Hence, the analysis of the correlation between *θ_i_*_0_ and *θ_iar_* is identified as the faulty phase position indicator. Similarly, the same correlation can be analyzed for faulty phase position identification if the fault occurs in phase ‘*b*’ and phase ‘*c*’. The required phase angles are extracted as follows:

From (14), three-phase rotor currents can be defined as follows:

(22) $$\left\{\begin{array}{l} i_{ar}=I_{ar}\sin(\theta +\theta_{iar})\\ i_{br}=I_{br}\sin(\theta +\theta_{ibr})\\ i_{cr}=I_{cr}\sin(\theta +\theta_{icr}) \end{array}\right.$$

where *I_ar_*, *I_br_*, and *I_cr_* are the FC magnitudes, and *θ_iar_*, *θ_ibr_*, and *θ_icr_* are the IPAs of the FCs in three-phase currents in (22). Also, angles of phase ‘b’ and phase ‘c’ are *θ_ibr_* = *θ_iar_* − 2π/3 and *θ_icr_* = *θ_iar_* + 2π/3. Generalizing (18) and (19) for phase ‘*k*’, the FC in ZSC can be written as follows:

(23) $$\left\{\begin{array}{l} i_{0}=I_{0M}\sin(\theta +\theta_{ikr0})\\ \theta_{ikr0}=\theta_{ikrf}+\delta \end{array}\right.$$

where *k* = *a*, *b*, *c*; *θ_ikr_*_0_ is the IPA of the FC in ZSC when the fault occurs in phase ‘*k*’ of the rotor windings; and *θ_ikrf_* is the IPA of faulty phase current.

The difference between the IPA of the FC in ZSC and the IPA of the rotor-phase currents (*θ_ikr_*) is the position indicator. From (22) and (23), the position indicator for fault in phase ‘*k*’ can be defined as follows:

(24) $$d_{k0}=\left|\theta_{ikr0}-\theta_{ikr}\right|$$

where *d_k_*_0_ is the position indicator. Taking into account that *δ* in (23) is very small as compared to *θ_ikr__f_* and *θ_ikr_*, (24) can be rewritten as follows:

(25) $$d_{k0}=\left|\delta \right|+\left|\theta_{ikrf}-\theta_{ikr}\right|$$

#### Fault Position Indicator Under Dynamic Conditions

Under sub-synchronous and super-synchronous modes of operation of DFIG, the phase sequence of the rotor currents will be positive and negative, respectively. The analysis based on the changes in phase sequences under these two modes of operation is given further.

(1) Sub-synchronous mode

The phase sequence of the rotor three-phase currents under the DFIG sub-synchronous mode of operation is positive (abc phase sequence); therefore, (25) and the angles of phase ‘*b*’ and phase ‘*c*’ in (22) remain unchanged. Hence, if the ITSC fault occurs in phase ‘a’, the values of position indicator *d_k_*_0_ for the three phases under sub-synchronous mode can be determined from (25), as follows:

(26) $$\begin{array}{l} d_{a0}=\left|\delta \right|+\left|\theta_{iar}-\theta_{iar}\right|=\left|\delta +0\right|\approx \left|\delta \right|\\ d_{b0}=\left|\delta \right|+\left|\theta_{iar}-\theta_{ibr}\right|=\left|\delta \right|+\left|\theta_{iar}-(\theta_{iar}-2\pi /3)\right|=\left|\delta \right|+\left|2\pi /3\right|\approx \left|\delta +2\pi /3\right|\\ d_{c0}=\left|\delta \right|+\left|\theta_{iar}-\theta_{icr}\right|=\left|\delta \right|+\left|\theta_{iar}-(\theta_{iar}+2\pi /3)\right|=\left|\delta \right|+\left|-2\pi /3\right|\approx \left|\delta +2\pi /3\right| \end{array}$$

So, for the fault in phase ‘*a*’, the values of *d_k_*_0_ for phases ‘*a*’, ‘*b*’ and ‘*c*’ are |*δ*|, |*δ* + 2π/3|, and |*δ* + 2π/3|, respectively.

Similarly, for the fault in phase ‘*b*’, *d_k_*_0_ values for phases ‘*a*’, ‘*b*’, and ‘*c*’ are |*δ* + 2π/3|, |*δ*|, and |*δ* + 4π/3|, respectively, and for the fault in phase ‘*c*’, these values are |*δ* + 2π/3|, |*δ* + 4π/3|, and |*δ*|, respectively. Table 2 summarizes these values.

It can be seen from Table 2 that *d*_*k*0_ has the smallest value for the faulty phase when the fault occurs in any of the three phases. Hence, the rotor phase with the smallest *d*_*k*0_ value is identified as the faulty phase when DFIG operates under sub-synchronous mode, where *d*_*k*0_ is the difference between the IPA of the FC in ZSC and the IPA of the FC in one of the rotor three-phase currents.

(2) Super-synchronous mode

The phase sequence of the rotor three-phase currents of DFIG under super-synchronous mode of operation is negative (acb phase sequence); therefore, *θ*_*ikr*0_ is negative and (25) takes the following form:

(27) $$s_{k0}=\left|-\theta_{ikr0}-\theta_{ikr}\right|=\left|\theta_{ikr0}+\theta_{ikr}\right|=\left|\delta \right|+\left|\theta_{ikrf}+\theta_{ikr}\right|$$

where *s_k_*_0_ is the position indicator for the super-synchronous mode. Also, for the negative phase sequence, phase ‘*b*’ and phase ‘*c*’ angles in (22) are *θ_ibr_* = *θ_iar_* + 2π/3 and *θ_icr_* = *θ_iar_* − 2π/3. Hence, if the ITSC fault occurs in phase ‘*a*’, the values of position indicator *s_k_*_0_ for the three phases under super-synchronous mode can be determined from (27), as follows:

(28) $$\begin{array}{l} s_{a0}=\left|\delta \right|+\left|\theta_{iar}+\theta_{iar}\right|=\left|\delta \right|+\left|2\theta_{iar}\right|\approx \left|\delta \right|\\ s_{b0}=\left|\delta \right|+\left|\theta_{iar}+\theta_{ibr}\right|=\left|\delta \right|+\left|\theta_{iar}+(\theta_{iar}+2\pi /3)\right|=\left|2\theta_{iar}\right|+\left|\delta \right|+\left|2\pi /3\right|\approx \left|\delta +2\pi /3\right|\\ s_{c0}=\left|\delta \right|+\left|\theta_{iar}+\theta_{icr}\right|=\left|\delta \right|+\left|\theta_{iar}+(\theta_{iar}-2\pi /3)\right|=\left|2\theta_{iar}\right|+\left|\delta \right|+\left|-2\pi /3\right|\approx \left|\delta +2\pi /3\right| \end{array}$$

where 2*θ_iar_* has an equal contribution in all three phases. So, for the fault in phase ‘*a*’, the values of *s_k_*_0_ for phases ‘*a*’, ‘*b*’, and ‘*c*’ are |*δ*|, |*δ* + 2π/3|, and |*δ* + 2π/3|, respectively. Similarly, for the fault in phase ‘*b*’, the *s_k_*_0_ values for phases ‘*a*’, ‘*b*’, and ‘*c*’ are |*δ* + 2π/3|, |*δ* + 4π/3|, and |*δ*|, and for the fault in phase ‘c’, the *s_k_*_0_ values are |*δ* + 2π/3|, |*δ*|, and |*δ* + 4π/3|, respectively. Table 3 summarizes these values.

It can be seen from Table 3 that *s_k_*_0_ has the smallest value for phase ‘*a*’, phase ‘*b*’, and phase ‘c’ when ITSC fault occurs in phase ‘*a*’, phase ’*c*’, and phase ’*b*’, respectively. Hence, under super-synchronous mode, phase ‘*a*’, phase ‘*b*’, and phase ‘*c*’ are identified as faulty phases when *s*_*a*0_, *s*_*c*0_, and *s*_*b*0_ are the smallest, respectively, where *s*_*k*0_ is the sum of IPA of the FC in ZSC and the IPA of the FC in one of the rotor three-phase currents.

### 3.4. Algorithm for Rotor ITSC Fault Diagnosis

**Figure 4:**
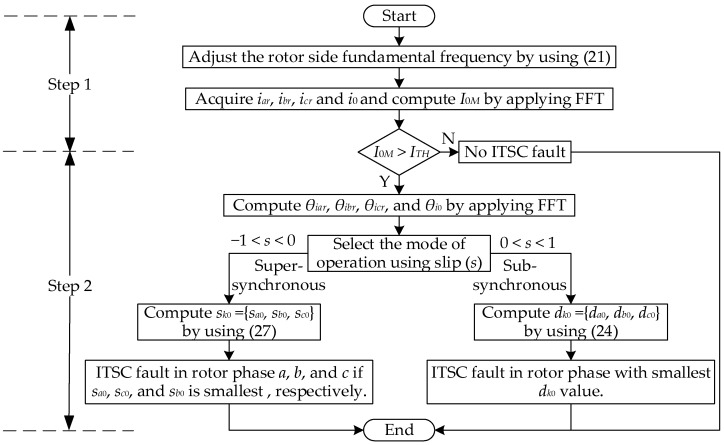
Online rotor ITSC fault diagnosis algorithm for DFIG.

Figure 4 shows the algorithm for implementing the comprehensive methodology for online rotor ITSC fault diagnosis of the DFIG. Detailed steps of the online diagnosis process are as follows:

Step 1: The algorithm starts with the measurement of rotor-side quantities, including phase currents and rotor speed. Rotor-side fundamental frequency is adjusted based on (21). The online extraction of the FC magnitude in ZSC (*I*_0*M*_) is carried out using FFT, and *I*_0*M*_ is compared with its pre-set threshold value obtained under healthy operating conditions (*I_TH_*). If *I*_0*M*_ exceeds *I_TH_*, it indicates the occurrence of an ITSC fault in one of the rotor phases. Conversely, if *I*_0*M*_ is less than *I_TH_*, it indicates a healthy rotor winding. The extent to which the *I*_0*M*_ exceeds *I_TH_* reflects the fault severity level.

Step 2: After receiving the signal of ITSC fault occurrence, the faulty phase is identified in the next step. Online extraction of IPA of the FC in ZSC (*θ*_*i*0_) and IPAs of the FCs in three-phase rotor currents (*θ_iar_*, *θ_ibr_*, and *θ_icr_*) are obtained using FFT. The mode of operation of DFIG is selected based on the value of the slip *s*. Positive slip indicates sub-synchronous while negative slip indicates super-synchronous mode of operation. The *d*_*k*0_ values are computed using (25) if the DFIG operates under sub-synchronous mode, and the rotor phase with the smallest *d*_*k*0_ value is identified as the faulty phase. On the other hand, the *s*_*k*0_ values are computed using (27) if the DFIG operates under super-synchronous mode, and the rotor phase *a*, *b*, and *c* is identified as the faulty phase if *s*_*a*0_, *s*_*c*0_, and *s*_*b*0_ is the smallest among *s*_*k*0_ values, respectively.

## 4. Simulations

To validate the capability of the proposed methodology for effective and reliable rotor ITSC fault diagnosis, simulations of the DFIG model have been carried out in MATLAB/Simulink. A short circuit of possibly minimum turns in the winding manifests early-stage fault with low severity level. Therefore, the simulations have been performed considering the healthy condition of the DFIG with no ITSC and the faulty condition with single-turn, two-turn, and five-turn short circuits in the rotor winding under variable load and variable speed conditions, manifesting its sub-synchronous and super-synchronous modes of operation. Table 4 enlists the parameter details of the simulated DFIG. Also, the simulation considers ITSC fault only in phase ‘*a*’ of the rotor winding as an example case for the proposed fault diagnosis methodology validation.

It is important to mention here that DFIG slip significantly influences the detectability of rotor ITSC faults. Higher slip amplifies fault-related effects, making detection easier, while low slip suppresses these effects, posing challenges [46]. Also, maintaining a narrow slip range between a negative value in super synchronous mode and a positive value in sub-synchronous mode is sometimes crucial for the DFIG in real-time applications to effectively cooperate with the power grid and operate close to synchronous speed [47]. Therefore, to ensure the realistic variable operating conditions of DFIG under such circumstances, the simulations have considered slip and load combinations within the slip range of (±0.1–±10%) and load range of (50–100%).

### 4.1. Simulation Under Sub-Synchronous Mode of Operation

In order to incorporate variable operating conditions (OCs) under sub-synchronous mode of operation, simulations consider the following three scenarios, each exhibiting variable speed and variable load operation conditions of the DFIG.

- OC1: DFIG operation with slip *s* = 0.03 and load= 100%.
- OC2: DFIG operation with slip *s* = 0.05 and load= 75%.
- OC3: DFIG operation with slip *s* = 0.1 and load= 50%.

OC1 represents a positive low-slip and full-load realistic scenario. This scenario indicates that the DFIG is operating near synchronous speed while delivering its maximum output. This scenario generally occurs at peak wind speeds, enabling optimal power extraction while maintaining high efficiency.

OC2 represents a positive low-slip and high-load realistic scenario. This scenario indicates that the DFIG is operating slightly further from synchronous speed while delivering 75% of its rated output. Such conditions typically arise under moderate wind speeds, where the generator maintains efficient power conversion while operating below maximum capacity.

OC3 represents a positive low-slip and low-load realistic scenario. This scenario indicates that the DFIG is running at a slightly higher slip while delivering half of its rated output. This scenario commonly occurs at lower wind speeds, where the power extracted is reduced, but the generator continues to function efficiently within its operating range.

**Figure 5:**
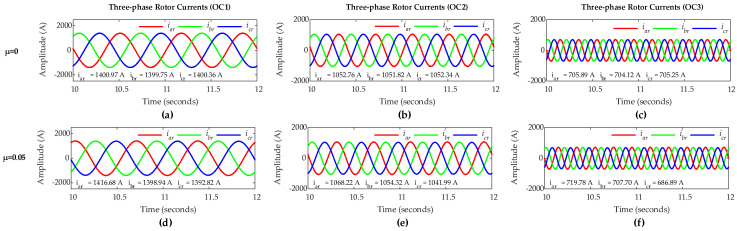
Three-phase rotor currents of DFIG: healthy rotor winding under (**a**) OC1, (**b**) OC2, and (**c**) OC3; five-turn short circuit fault in phase-a of rotor winding under (**d**) OC1, (**e**) OC2, and (**f**) OC3.

Simulation of the DFIG is carried out considering the sub-synchronous mode with *µ* = 0 for healthy rotor winding and *µ* = 0.05 for rotor winding representing a five-turn short circuit fault in phase ‘*a*’. Figure 5 shows three-phase rotor currents for healthy and faulty rotor winding conditions under OC1, OC2, and OC3 scenarios. The positive phase sequence (abc-phase sequence) of the rotor currents confirms that the DFIG operates under sub-synchronous mode. For healthy condition with *µ* = 0, three-phase rotor currents are nearly symmetrical across all three scenarios, whereas, for faulty condition with *µ* = 0.05, three-phase rotor currents only carry an insignificant asymmetry even under a relatively high severity fault case.

Therefore, analyzing the rotor three-phase current waveforms in the time domain does not provide sufficient clarity to detect and locate ITSC faults in rotor windings, especially at a low severity stage. Hence, to address this, FCs in three-phase rotor current signals and ZSC signal are analyzed using FFT for the effective fault diagnosis.

**Figure 6:**
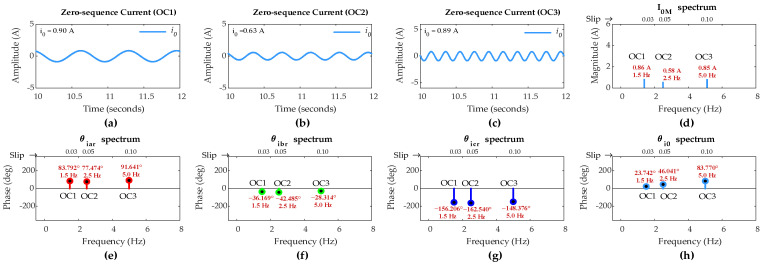
Simulation results of DFIG with healthy rotor winding: zero-sequence current under (**a**) OC1, (**b**) OC2, and (**c**) OC3; (**d**) *I*_0*M*_ under OC1–OC3; (**e**) *θ_iar_* under OC1–OC3; (**f**) *θ_ibr_* under OC1–OC3; (**g**) *θ_icr_* under OC1–OC3; (**h**) *θ_i_*_0_ under OC1–OC3.

Figure 6 shows ZSC waveforms and FFT results for the healthy rotor winding with *µ* = 0 under OC1, OC2, and OC3 scenarios. Since the DFIG operates at different slips in three scenarios, the rotor-side fundamental frequency should be different for each scenario according to (21). FFT results show that the rotor-side fundamental frequency for OC1, OC2, and OC3 is 1.5 Hz, 2.5 Hz, and 5 Hz, respectively, aligning perfectly with (21).

Ideally, ZSC should be zero for healthy winding; however, a small amount of ZSC is present in all three scenarios. FFT results show that the FC magnitude in ZSC for healthy rotor winding under OC1, OC2, and OC3 is 0.86 A, 0.58 A, and 0.85 A, respectively.

Hence, for the inclusion of imperfections in the machine operation, including a small imbalance in the DIFG rotor currents and the measurement noise, the threshold value of FC magnitude in ZSC (*I_TH_*) has been set at 1.2 A. As per the proposed algorithm, any FC magnitude in ZSC exceeding this pre-set threshold will indicate an ITSC fault in the rotor winding.

**Figure 7:**
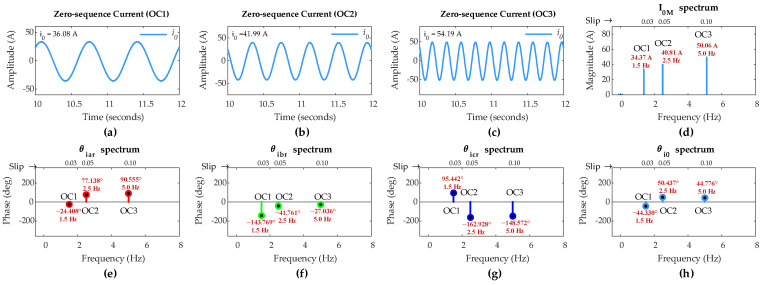
Simulation results of DFIG with five-turn fault in phase-a of rotor winding: zero-sequence current under (**a**) OC1, (**b**) OC2, and (**c**) OC3; (**d**) *I*_0*M*_ under OC1–OC3; (**e**) *θ_iar_* under OC1–OC3; (**f**) *θ_ibr_* under OC1–OC3; (**g**) *θ_icr_* under OC1–OC3; (**h**) *θ_i_*_0_ under OC1–OC3.

Figure 7 shows the ZSC waveforms and the FFT results for a five-turn short circuit fault in phase ‘a’ under OC1, OC2, and OC3 operational condition scenarios. Based on 100 turns in rotor winding, the fault severity level *µ* is 0.05. The ZSC is significantly higher and prominent due to the asymmetry in three-phase rotor currents caused by the five-turn fault, as shown in Figure 5.

Further FFT analysis of the rotor currents and ZSC signals identifies that the FC magnitude in ZSC for five-turn fault is 34.37 A under OC1, 40.81 A under OC2, and 50.06 A under OC3. The IPA of the FC in ZSC is −44.33° under OC1, 50.44° under OC2, and 44.78° under OC3. The IPAs of the FCs in rotor currents for phases a, b, and c are −24.41°, −143.77°, and 95.44°, respectively, under OC1; 77.14°, −41.76°, and −162.93°, respectively, under OC2; and 90.56°, −27.04°, and −148.57°, respectively, under OC3.

Considering step 1 of the proposed algorithm, since the FC magnitude in ZSC exceeds pre-set threshold value of 1.2 A under all three scenarios, this indicates the occurrence of an ITSC fault in the rotor winding of DFIG under sub-synchronous mode and variable operating conditions.

Considering step 2 of the algorithm, (24) computes *d*_*k*0_ values to identify the rotor winding phase carrying ITSC fault under sub-synchronous mode. The computed values of *d*_*a*0_, *d*_*b*0_, and *d*_*c*0_ are 19.92°, 99.44°, and 139.77°, respectively, under OC1; 26.70°, 92.20°, and 213.37°, respectively, under OC2; and 45.78°, 71.81°, and 193.35°, respectively, under OC3. Since *d*_*a*0_ is the smallest value among the *d*_*k*0_ values in all three scenarios, the proposed algorithm accurately identifies ITSC fault occurrence in phase ‘a’ under sub-synchronous mode and variable operating conditions.

However, a five-turn fault might be considered as a relatively high severity fault compared to two-turn and single-turn faults. Therefore, the methodology is further applied to relatively low-severity ITSC faults for validation under sub-synchronous mode.

**Figure 8:**
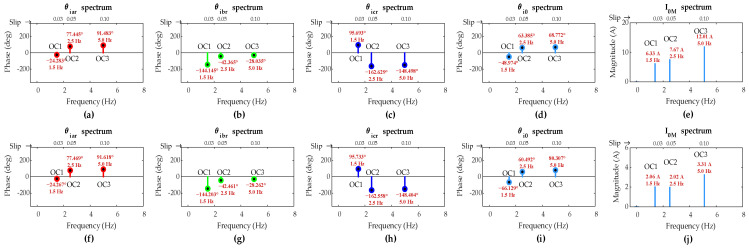
Simulation results of DFIG under OC1-OC3 scenarios with rotor winding: two-turn fault in phase-a (**a**) *θ_iar_*; (**b**) *θ_ibr_*; (**c**) *θ_icr_*; (**d**) *θ_i_*_0_; (**e**) *I*_0*M*_; single-turn fault (**f**) *θ_iar_*; (**g**) *θ_ibr_*; (**h**) *θ_icr_*; (**i**) *θ_i_*_0_; (**j**) *I*_0*M*_.

Figure 8 shows FFT results of rotor currents and the ZSC signals for two-turn short circuit with *µ* = 0.02 and single-turn short circuit with *µ* = 0.01 in phase ‘a’ of the rotor winding under OC1, OC2, and OC3 scenarios. The values of *µ* manifest lower severity faults than the fault in the previous case.

The FFT results reveal that the FC magnitude in ZSC for two-turn fault under all three scenarios is lower than that in previous case for five-turn fault, and for single-turn fault, it is even lower under the same scenarios. This confirms the direct dependency of FC magnitude in ZSC on fault severity level *µ*.

Table 5 summarizes the simulation results when DFIG operates under sub-synchronous mode. It is evident that the FC magnitude in ZSC for both two-turn and single-turn short circuits is greater than the pre-set threshold value of 1.2 A under all three scenarios, confirming the occurrence of fault even with low-severity ITSC in the rotor winding.

The *d*_*k*0_ values are computed using the IPA values of FCs in rotor currents and the ZSC, and they are given in Table 5. It can be found that *d*_*a*0_ is the smallest among the *d*_*k*0_ values in all three scenarios for two-turn and single-turn faults, validating the proposed algorithm’s accuracy in detecting ITSC fault in phase ‘*a*’ of the rotor winding, even at low severity levels under variable operating conditions.

### 4.2. Simulation Under Super-Synchronous Mode of Operation

In order to incorporate more distinct variable operating conditions (OCs), simulations consider the following three scenarios under the super-synchronous mode. Each scenario exhibits variable speed and load operation conditions of the DFIG.

- OC4: DFIG operation with slip *s* = −0.02 and load = 60%.
- OC5: DFIG operation with slip *s* = −0.06 and load = 75%.
- OC6: DFIG operation with slip *s* = −0.09 and load = 100%.

OC4 represents a negative low-slip and low-load realistic scenario. This scenario indicates that the DFIG is operating slightly above synchronous speed while delivering 60% of its rated output. This condition typically occurs at moderately high wind speeds, where the generator extracts a significant amount of power while maintaining stability.

OC5 represents a negative low-slip and high-load realistic scenario. This scenario indicates that the DFIG is running further into the super-synchronous region while supplying 75% of its rated output. Such conditions are common when wind speeds are relatively high, allowing the generator to operate efficiently while feeding more power into the grid.

OC6 represents a negative low-slip and full-load realistic scenario. This scenario indicates that the DFIG is operating slightly higher above the synchronous speed while delivering its maximum output. This scenario typically arises at very high wind speeds, enabling the generator to achieve peak power extraction while utilizing its full capacity.

**Figure 9:**
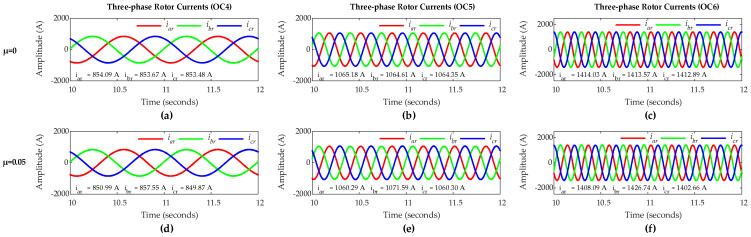
Three-phase rotor currents of DFIG: healthy rotor winding under (**a**) OC4, (**b**) OC5, and (**c**) OC6; five-turn short circuit fault in phase-a of rotor winding under (**d**) OC4, (**e**) OC5, and (**f**) OC6.

Considering the super-synchronous mode, simulation of DFIG is carried out with µ = 0 for healthy rotor winding and *µ* = 0.05 for rotor winding with a five-turn short circuit fault in phase ‘*a*’. Figure 9 shows three-phase rotor currents with healthy and faulty rotor winding conditions under OC4, OC5, and OC6 scenarios. The negative phase sequence (acb-phase sequence) of the rotor currents in all three scenarios manifests DFIG operation under super-synchronous mode. In all three scenarios, rotor currents are nearly symmetrical for healthy rotor winding with *µ* = 0, whereas for faulty condition with *µ* = 0.05, rotor currents carry an insignificant asymmetry which is not prominent even under a relatively high-severity fault case. Hence, also in super-synchronous mode, FCs in three-phase rotor currents and ZSC are analyzed using FFT for the effective fault diagnosis.

**Figure 10:**
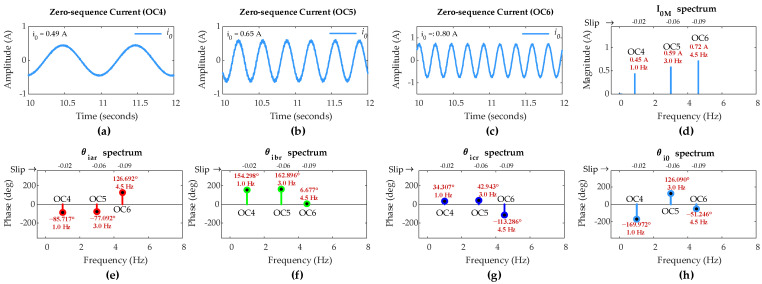
Simulation results of DFIG with healthy rotor winding: zero-sequence current under (**a**) OC4, (**b**) OC5, (**c**) and OC6; (**d**) *I*_0*M*_ under OC4–OC6; (**e**) *θ_iar_* under OC4–OC6; (**f**) *θ_ibr_* under OC4–OC6; (**g**) *θ_icr_* under OC4–OC6; (**h**) *θ_i_*_0_ under OC4–OC6.

Figure 10 shows the ZSC waveforms and the FFT results for healthy rotor winding conditions with µ = 0, under OC4, OC5, and OC6 scenarios. Since DFIG operates at different a slip in each of three scenarios, the rotor-side fundamental frequency should be different for each scenario according to (21). FFT results reveal the distinct fundamental frequencies: 1 Hz for OC4, 3 Hz for OC5, and 4.5 Hz for OC6, which are exactly in accordance with (21). Ideally, ZSC should be absent in healthy rotor winding. However, a small amount of ZSC shows up in all three scenarios on account of the minor imperfections in the machine operation.

The FC magnitude in ZSC for healthy rotor winding under OC4, OC5, and OC6 is 0.45 A, 0.59 A, and 0.72 A, respectively. Hence, to compensate for the imperfections in the machine, the threshold value of FC magnitude in ZSC (*I_TH_*) is again set to 1.2 A. Again, as outlined in the proposed algorithm, any FC magnitude in ZSC exceeding this pre-set threshold will be the indication of an ITSC fault in the rotor winding.

**Figure 11:**
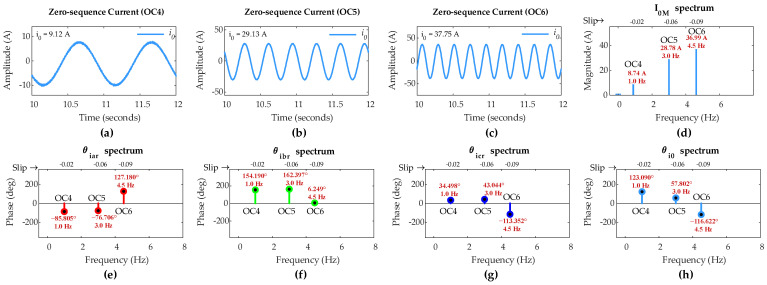
Simulation results of DFIG with five-turn fault in phase-a of rotor winding: zero-sequence current under (**a**) OC4, (**b**) OC5, and (**c**) OC6; (**d**) *I*_0*M*_ under OC4–OC6; (**e**) *θ_iar_* under OC4–OC6; (**f**) *θ_ibr_* under OC4–OC6; (**g**) *θ_icr_* under OC4–OC6; (**h**) *θ_i_*_0_ under OC4–OC6.

Figure 11 shows the ZSC waveforms and the FFT results for a five-turn short circuit fault in phase ‘a’ of the rotor winding with µ = 0.05, under OC4, OC5, and OC6 scenarios. The ZSC is significantly higher and prominent due to the asymmetry in three-phase rotor currents caused by the five-turn fault, as shown in Figure 9.

Application of FFT on the rotor currents and the ZSC signals in this case discloses that the FC magnitude in ZSC is 8.74 A under OC4, 28.78 A under OC5, and 36.99 A under OC6. Under these scenarios, the IPA of the FC in ZSC is 123.09°, 57.80°, and −116.62°, respectively.

The IPAs of the FCs in rotor currents for phases a, b, and c are −85.81°, 34.50°, and 154.19°, respectively, under OC4; −76.71°, 43.04°, and 162.40°, respectively, under OC5; and 127.18°, −113.35°, and 6.25°, respectively, under OC6.

As per step 1 of the proposed algorithm, since the FC magnitude in ZSC for five-turn short circuit fault is greater than the pre-set threshold value of 1.2 A under all three scenarios, this indicates the occurrence of an ITSC fault in the rotor winding of DFIG under super-synchronous mode and variable operating conditions.

As per step 2 of the algorithm, (27) computes *s*_*k*0_ values for identifying the rotor faulty phase under super-synchronous mode. The computed *s*_*a*0_, *s*_*b*0_, and *s*_*c*0_ values are 37.29°, 157.59°, and 277.28°, respectively, under OC4; 18.90°, 100.85°, and 220.20°, respectively, under OC5; and 10.56°, 229.97°, and 110.35°, respectively, under OC6. Since *s*_*a*0_ is the smallest among the *s*_*k*0_ values in all three scenarios, the proposed algorithm accurately identifies phase ‘a’ as the faulty rotor winding phase of DFIG under super-synchronous mode and variable operating conditions.

Yet again, as the five-turn short circuit is relatively a high-severity ITSC fault as compared to two-turn and single-turn faults, the methodology is further applied to relatively low-severity ITSC faults for validation under super-synchronous mode of operation.

**Figure 12:**
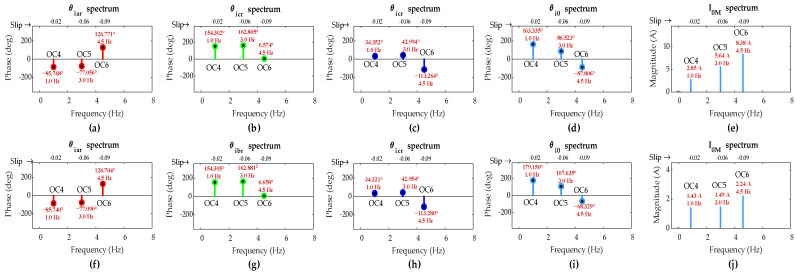
Simulation results of DFIG under OC4–OC6 scenarios with rotor winding: two-turn fault in phase-a (**a**) *θ_iar_*, (**b**) *θ_ibr_*, (**c**) *θ_icr_* (**d**) *θ_i_*_0_, and (**e**) *I*_0*M*_; single-turn fault (**f**) *θ_iar_*, (**g**) *θ_ibr_*, (**h**) *θ_icr_*, (**i**) *θ_i_*_0_, and (**j**) *I*_0*M.*_

Figure 12 shows FFT results of rotor three-phase currents and the ZSC signals for two-turn short circuit fault with *µ* = 0.02 and single-turn short circuit fault with *µ* = 0.01 in phase ‘a’ of the rotor winding under OC4, OC5, and OC6 scenarios. The values of *µ* manifest relatively lower severity faults than the fault in the previous case.

The FFT results show that the FC magnitude in ZSC for two-turn fault is lower compared to the previous case for five-turn fault across all OC4-OC6 scenarios. Also, FC magnitude in ZSC for single-turn fault is even lower than that observed for two-turn fault under the same scenarios, clearly revealing the dependency of FC magnitude in ZSC on fault severity level μ.

Table 6 summarizes the simulation results when DFIG operates under super-synchronous mode, considering OC4, OC5, and OC6 scenarios. It can be seen that the FC magnitude in ZSC for two-turn and single-turn short circuit faults across all three scenarios exceeds the pre-set threshold value of 1.2 A, which indicates the occurrence of fault even with low-severity ITSC in the rotor winding.

The *s*_*k*0_ values are computed in Table 6. Evidently, *s*_*a*0_ is the smallest among the *s*_*k*0_ values in all three scenarios for two-turn and single-turn faults, validating that the proposed algorithm accurately identifies the occurrence of fault in phase ‘*a*’ of the rotor winding under variable operating conditions even in low-severity ISTC fault situations when DFIG operates in super-synchronous mode.

It can be noticed from Figure 8, Figure 9, Figure 11 and Figure 12 that the load and speed affect the ZSC values at different fault levels, providing insight into how load and speed variation impact the detection of ITSC in the DFIG. However, the methodology successfully detects and locates the ITSC fault under all these variable conditions, ensuring robust fault detection despite fluctuations in load and speed.

### 4.3. Comparitive Analysis with the Existing Method

#### 4.3.1. Comparative Analysis of Fault Indicator (I_0M_)

**Figure 13:**
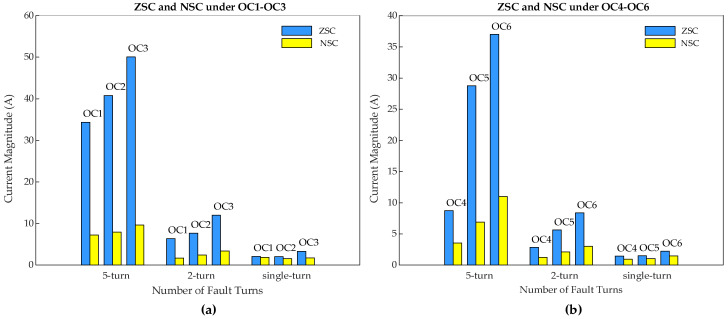
Comparative analysis of proposed ZSC-based fault indicator (*I*_0*M*_) with the existing NSC-based fault indicator for five-turn, two-turn, and single-turn rotor winding ITSC in phase-a under (**a**) OC1-OC3 scenarios and (**b**) OC4-OC6 scenarios.

A comparative analysis between the proposed ZSC-based fault indicator (*I*_0*M*_) and the existing negative sequence current (NSC)-based fault indictor is provided in Figure 13. It can be seen that, in sub-synchronous mode, ZSC values for five-turn fault are 34.37 A, 40.81 A, and 50.06 A under OC1, OC2, and OC3, respectively, which are much higher compared to NSC values of 12.85 A, 16.32 A, and 20.45 A, in the respective cases.

Even under low-severity ITSC faults, ZSC shows higher values compared to NSC. For two-turn ITSC, ZSC is 6.33 A, 7.67 A, and 12.01 A under OC1, OC2, and OC3, respectively, whereas NSC remains lower at 2.06 A, 2.53 A, and 3.91 A, respectively. Similarly, for single-turn ITSC, ZSC values are 2.06 A, 2.02 A, and 3.31 A under OC1, OC2, and OC3, respectively, while NSC values are only 0.89 A, 1.53 A, and 1.78 A, respectively.

A similar pattern is observed in super-synchronous mode. For five-turn fault, ZSC values are 8.74 A, 28.78 A, and 36.99 A under OC4, OC5, and OC6, respectively, which are much higher than NSC values of 3.54 A, 6.89 A, and 11.02 A under the respective cases. The difference remains significant even for two-turn fault, where ZSC values reach 2.85 A, 5.64 A, and 8.38 A, compared to 1.25 A, 2.12 A, and 3.01 A for NSC. For single-turn fault, ZSC values are 1.43 A, 1.49 A, and 2.24 A, while NSC shows lower values of 0.95 A, 1.02 A, and 1.47 A.

This confirms that NSC alone may fail to provide a clear fault indication, particularly for early-stage ITSC faults with low severity levels, whereas ZSC is far more sensitive and reliable than NSC which makes it a more effective indicator for ITSC fault detection in DFIG rotor windings regardless of its operating mode under variable speed and load conditions.

#### 4.3.2. Comparative Analysis of Fault Location Indicators (d_k0_ and s_k0_)

**Figure 14:**
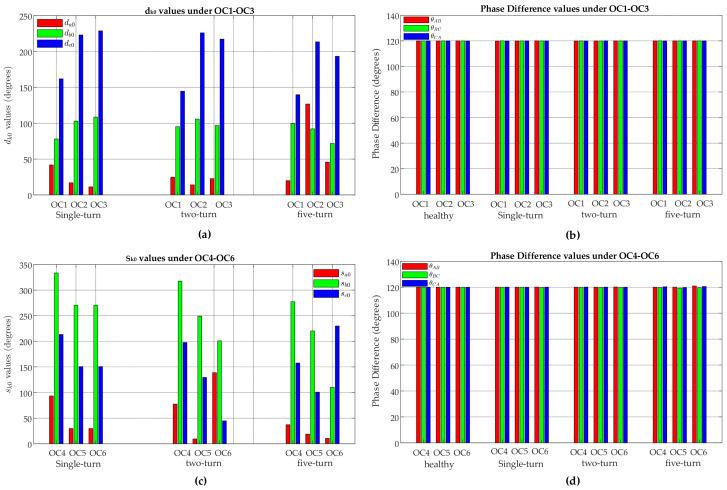
Comparative analysis of proposed ZSC-based fault location indicators *d*_*k*0_ and *s*_*k*0_ with the existing phase difference-based fault location indicator for five-turn, two-turn, and single-turn rotor winding ITSC in phase-a (**a**) *d*_*k*0_ values under OC1–OC3 scenarios; (**b**) phase-difference values under OC1–OC3; (**c**) *s*_*k*0_ values under OC4-OC6 scenarios; and (**d**) phase-difference values under OC4–OC6 scenarios.

A comparative analysis between the proposed ZSC-based fault location indicators (*d*_*k*0_ and *s*_*k*0_) and the existing phase difference-based fault location indicator is given in Figure 14. It can be seen that, in sub-synchronous mode, *d*_*k*0_ values (*d*_*a*0_, *d*_*b*0_, and *d_c_*_0_) for five-turn, two-turn, and single-turn faults show significant variations across all OC1–OC3 scenarios. On the other hand, the phase-difference values (*θ_ab_*, *θ_bc_*, and *θ_ca_*) of the three-phase rotor currents under the corresponding fault and operating condition scenarios remain consistently close to 120°, ranging between 119° and 121°. Even under the high-level fault of five turns, phase-difference shows a minimal deviation of just 0.07°, which is negligibly low compared to the deviations observed in *d*_*k*0_ values.

A similar trend is observed in super-synchronous mode. The *s*_*k*0_ values (*s_a_*_0_, *s*_*b*0_, and *s_c_*_0_) for five-turn, two-turn, and single-turn faults fault show significant variations across all OC4–OC6 scenarios, whereas the phase-difference values (*θ_ab_*, *θ_bc_*, and *θ_ca_*) of the three-phase rotor currents under the corresponding fault and operating conditions scenarios remain nearly close to 120°, ranging between 119° and 121°. Even under the high-level fault of five turns, phase-difference shows a minimal deviation of just 0.9°, which is negligibly low compared to the deviations observed in *s*_*k*0_ values.

This shows that while phase-difference values remain almost unaffected, regardless of fault severity or operating conditions, the *d_k_*_0_ and *s*_*k*0_ values are highly sensitive to these factors. As a result, *d*_*k*0_ and *s*_*k*0_ are much more effective for identifying ITSC fault locations in DFIG rotor windings compared to phase-difference-based indicator.

Also, it is worth noting the following from the simulation results:

- The FC magnitude in ZSC increases as the fault severity level *µ* increases and decreases as the value of *µ* decreases, under all OC1–OC6 scenarios.
- IPAs of the FCs in rotor three-phase currents are only a little affected by the fault severity level *µ*, whereas IPA of the FC in ZSC is greatly affected by the value of *µ* under all OC1–OC6 scenarios.
- Hence, ZSC offers high sensitivity to the severity and location of fault which makes it a reliable feature for low-severity fault detection under variable operating conditions.

## 5. Discussion

Since the ITSC fault is detected and the faulty phase is accurately identified in the simulation results under all OC1–OC6 scenarios with different fault severity levels, it is obvious that the proposed methodology is successfully validated and can be applied for online DFIG rotor winding ITSC fault detection and faulty phase identification under variable load and speed operating conditions. The proposed methodology is straightforward yet effective, and it notably involves simpler and less complicated signal processing in contrast to the other signal processing-based methods, saving time and computational resources.

The methodology is designed to handle real-world conditions where noise and interference are common. Specifically, it focuses on the fundamental component (FC) of the rotor’s three-phase currents and the zero-sequence current (ZSC), which is both large and highly sensitive to ITSC faults. The FC is extracted using Fast Fourier transform (FFT), a technique that efficiently filters out higher-order harmonics and measurement noise. By isolating the FC, which is the most relevant signal for fault detection, the influence of noise can be significantly reduced, ensuring that the fault detection process remains both accurate and reliable. Moreover, FFT is particularly effective at isolating the dominant frequency components related to faults while eliminating unwanted high-frequency noise and harmonics, which typically arise in practical scenarios due to various factors such as electrical equipment or environmental conditions. As a result, the methodology performs well even in the presence of typical interference and noise in real-world data, maintaining high fault detection accuracy.

Although the simulations validate the feasibility of the proposed methodology, employing it for real-time monitoring can present some challenges. Noise and interferences in the current signals complicate the analysis that can mask the faults in real-time applications. Imperfections in the manufacturing and measuring equipment can degrade the quality of the data that can impact the reliability of the methodology. Delays in the processing of the signals can slow down the response of fault indicators that can delay the remedial actions. To effectively address such challenges, technological improvements need to be integrated with the DFIG system to reduce the noise and inference in the signals and enhance the data quality and processing.

## 6. Conclusions

This paper proposes a ZSC-based comprehensive methodology for the rotor ITSC fault diagnosis of DFIG under variable operating conditions. Analytical evaluation of the DFIG mathematical model is conducted to identify the fault features. The FC magnitude in ZSC signal is identified as the fault indicator, and the relationship between the IPA of the FC in ZSC and the IPAs of the FCs in rotor three-phase currents is identified as the faulty phase location indicator. A simple yet highly reliable algorithm is presented to implement the proposed fault detection methodology. The DFIG model with ITSC faults of different severity levels in rotor winding is implemented, and extensive simulations, considering the variable operating conditions under both sub-synchronous and super-synchronous modes of DFIG operation, are carried out using MATLAB/Simulink environment. The simulation results confirm that the proposed methodology is capable of effectively detecting ITSC faults at a low-severity stage and precisely identifying the faulty phase within the rotor windings of the DFIG. The summary of the research highlights presented in this paper is provided below:

(1) While previous research has largely overlooked the super-synchronous mode and rotor-side dynamic low-frequency conditions, the proposed methodology remains effective for rotor ITSC fault detection and faulty phase identification under both sub-synchronous and super-synchronous modes of DFIG operation with rotor-side dynamic low-frequency conditions, ensuring its reliability in practical applications.
(2) The FC magnitude in ZSC signal is large and highly sensitive to ITSC fault severity levels in DFIG rotor windings, enabling the effective fault detection even at an early stage with low-severity level. However, ZSC signal is not inherently available, and an auxiliary arrangement is required on the rotor side. As a result, this approach is most appropriate for large-scale and high-cost DFIG applications.
(3) IPA of the FC in ZSC is highly sensitive to ITSC fault severity levels and the faulty phase location in DFIG rotor windings. On the other hand, IPAs of the FCs in rotor three-phase currents are only a little sensitive to faults. Therefore, the correlation between these angles is effective for the faulty phase location. When the DFIG operates under the sub-synchronous mode, rotor three-phase currents have positive phase sequence (abc), and the difference between the IPA of the FC in ZSC and the IPA of the FC in rotor faulty phase current (d_*k*0_) is the smallest angle. Hence, *d*_*a*0_, *d*_*b*0_, and *d*_*c*0_ are the smallest values when an ITSC occurs in rotor phase ‘a’, ‘b’, and ‘c’, respectively. However, when the DFIG operates under the super-synchronous mode, rotor three-phase currents have the negative phase sequence (acb), and the sum of the IPA of the FC in ZSC and the IPA of the FC in rotor-phase current (*s*_*k*0_) is the smallest angle for phases ‘a’, ‘b’, and ‘c’ when the fault occurs in phases ‘a’, ‘c’, and ‘b’, respectively. Hence, *s*_*a*0_, *s*_*b*0_, and *s*_*c*0_ are the smallest values when an ITSC occurs in rotor phases ‘a’, ‘c’, and ‘b’, respectively.

Future work can focus on experimentally validating the detection of rotor ITSC faults in DFIG using sequence component analysis. This can involve setting up a small-scale laboratory system with a prototype DFIG to simulate real-world fault scenarios. By leveraging AI-based algorithms and real-time data analytics, rotor currents can be monitored under various operating conditions. This practical approach will help evaluate how well the fault detection method performs in real-world situations, particularly in terms of accuracy, reliability, and its ability to detect faults at an early stage.

## Figures and Tables

**Table 1:** Summary of the existing methods and contributions of the proposed method.

Method	Machine	ITSC Winding	Analytical Evaluation	Fault Position	Super-Synch. Mode	Frequency
Stator current analysis [3,7,15]	DFIG	stator	No	No	No	Fixed
Rotor current analysis [12,14]	DFIG	stator	No	No	No	Fixed
Currents/torque signal analysis [21]	DFIG	rotor	No	No	No	Fixed
Vibration signal analysis [16]	DFIG	stator	Yes	No	No	Fixed
Stator reactive power analysis [20]	DFIG	stator	Yes	No	No	Fixed
winding temperature analysis [25,27]	IM	stator	No	No	No	Fixed
Embedded control signal analysis [23,24]	PMSM	stator	Yes	No	No	Fixed
Embedded control signal analysis [36,37]	DFIG	rotor	Yes	No	Yes	dynamic
NSC * analysis [8,13]	DFIG	stator	Yes	Yes	No	Fixed
ZSV ** analysis [38]	PMSM	stator	No	No	No	Fixed
ZSC/NSC analysis [40]	DFIG	stator	No	No	No	Fixed
ZSV/ZSC analysis [42]	PMSM	stator	Yes	Yes	No	Fixed
ZSC analysis (present study)	DFIG	rotor	Yes	Yes	Yes	dynamic

* NSC = negative sequence current; ** ZSV = zero-sequence voltage.

**Table 2:** Rotor ITSC fault position indicator when DFIG operates under sub-synchronous mode.

Mode	Faulty Phase	*d_k_*_0_ = {*d_a_*_0_, *d_b_*_0_, *d_c_*_0_}	Position Indicator
Sub-synchronous (1 > *s* > 0)	Phase ‘*a*’	{|*δ*|, |*δ* + 2π/3|, |*δ* + 2π/3|}	smallest *d_a_*_0_
	Phase ‘*b*’	{|*δ* + 2π/3|, |*δ*|, |*δ* + 4π/3|}	smallest *d_b_*_0_
	Phase ‘*c*’	{|*δ* + 2π/3|, |*δ* + 4π/3|, |*δ*|}	smallest *d_c_*_0_

**Table 3:** Rotor ITSC fault position indicator when DFIG operates under super-synchronous mode.

Mode	Faulty Phase	*s_k_*_0_ = {*s_a_*_0_, *s_b_*_0_, *s_c_*_0_}	Position Indicator
Super-synchronous (0 > *s* > −1)	Phase ‘*a*’	{|*δ*|, |*δ* + 2π/3|, |*δ* + 2π/3|}	smallest *s_a_*_0_
	Phase ‘*b*’	{|*δ* + 2π/3|, |*δ* + 4π/3|, |*δ*|}	smallest *s_c_*_0_
	Phase ‘*c*’	{|*δ* + 2π/3|, |*δ*|, |*δ* + 4π/3|}	smallest *s_b_*_0_

**Table 4:** Simulation parameters of the DFIG.

Property	Value	Property	Value
Rated power	2 MW	Stator resistance	0.00261 Ω
RMS stator voltage (L-L)	690 V	Rotor resistance	0.00292 Ω
RMS rotor voltage (L-L)	207 V	Magnetizing inductance	2.5 mH
RMS stator current	1760 A	Stator leakage inductance	87 µH
RMS rotor current	1420 A	Rotor leakage inductance	783 µH
Stator/rotor winding	Star/delta	Grid-side frequency	50 Hz
Synchronous speed	1500 r/min	Pole pairs	2
Stator-phase series turns	333	Rotor-phase series turns	100

**Table 5:** Summary of the simulation results under sub-synchronous mode.

*µ*	OC Scenario	Mag. and IPA of FC in ZSC		IPAs of FCs in Three-Phase Rotor Currents			*d_k_*_0_ = |*θ*_*ikr*0_ − *θ_ikr_*|			Diagnosis Result
		*I*_0*M*_ (A)	*θ*_*i*0_ (deg.)	*θ_iar_* (deg.)	*θ_ibr_* (deg.)	*θ_icr_* (deg.)	*d*_*a*0_	*d*_*b*0_	*d*_*c*0_	
0	OC1	0.86	23.74	83.80	−36.17	−156.21	*I*_0*M*_ < *I_TH_*			Healthy rotor winding
	OC2	0.58	46.04	77.47	−42.49	−162.54				
	OC3	0.85	83.78	91.64	−28.31	−148.38				
0.05	OC1	34.37	−44.33	−24.41	−143.77	95.44	19.92	99.44	139.77	*d*_*a*0_ is smallest; ITSC in phase ‘*a*’
	OC2	40.81	50.44	77.14	−41.76	−162.93	26.70	92.20	213.37	
	OC3	50.06	44.78	90.56	−27.04	−148.57	45.78	71.81	193.35	
0.02	OC1	6.33	−48.97	−24.28	−144.15	95.69	24.69	95.17	144.67	*d*_*a*0_ is smallest; ITSC in phase ‘*a*’
	OC2	7.67	63.39	77.45	−42.37	−162.63	14.06	105.75	226.01	
	OC3	12.01	68.77	91.48	−28.04	−148.50	22.71	96.81	217.27	
0.01	OC1	2.06	−66.13	−24.27	−144.20	95.73	41.86	78.07	161.86	*d*_*a*0_ is smallest; ITSC in phase ‘*a*’
	OC2	2.02	60.50	77.47	−42.46	−162.56	16.98	102.95	223.05	
	OC3	3.31	80.31	91.62	−28.26	−148.40	11.31	108.57	228.71	

**Table 6:** Summary of the simulation results under super-synchronous mode.

*µ*	OC Scenario	Mag. and IPA of FC in ZSC		IPAs of FCs in Three Phase Rotor Currents			*s*_*k*0_ = |*θ*_*ikr*0_ + *θ_ikr_*|			Diagnosis Result
		*I*_0*M*_ (A)	*θ*_*i*0_ (deg.)	*θ_iar_* (deg.)	*θ_ibr_* (deg.)	*θ_icr_* (deg.)	*s*_*a*0_	*s*_*b*0_	*s*_*c*0_	
0	OC4	0.45	−169.97	−85.72	154.30	34.31	*I*_0*M*_ < *I_TH_*			Healthy rotor winding
	OC5	0.59	126.09	−77.09	162.90	42.94				
	OC6	0.72	−51.25	126.69	6.68	−113.29				
0.05	OC4	8.74	123.09	−85.81	154.19	34.50	37.29	277.28	157.59	*s*_*a*0_ is smallest; ITSC in phase ‘*a*’
	OC5	28.78	57.80	−76.71	162.40	43.04	18.90	220.20	100.85	
	OC6	36.99	−116.62	127.18	6.25	−113.35	10.56	110.35	229.97	
0.02	OC4	2.85	163.34	−85.77	154.30	34.35	77.57	317.64	197.69	*s*_*a*0_ is smallest; ITSC in phase ‘*a*’
	OC5	5.64	86.52	−77.06	162.81	42.99	9.47	249.33	129.52	
	OC6	8.38	−87.81	126.77	6.57	−113.26	38.97	201.07	44.81	
0.01	OC4	1.43	179.15	−85.74	154.31	34.32	93.41	333.46	213.47	*s_a_*_0_ is smallest; ITSC in phase ‘*a*’
	OC5	1.49	107.63	−77.09	162.88	42.95	29.74	270.52	150.59	
	OC6	2.24	−68.33	126.70	6.58	−113.28	58.38	61.67	181.61	

## Data Availability

Data are contained within the article.

## Nomenclature

DFIG	Doubly fed induction generator
ITSC	Inter-turn short circuit
ZSC	Zero-sequence current
FFT	Fast Fourier transform
FC	Fundamental component
FCs	Fundamental components
IPA	Initial phase angle
IPAs	Initial phase angles
